# VIA/VILI is more suitable for cervical cancer prevention in Chinese poverty-stricken region: a health economic evaluation

**DOI:** 10.1186/s12889-017-4054-9

**Published:** 2017-01-25

**Authors:** Yu Xie, Xiaodong Tan, Haiyan Shao, Qing Liu, Jiyu Tou, Yuling Zhang, Qiong Luo, Qunying Xiang

**Affiliations:** 1 School of Public Health, Wuhan University, Hubei, China; 2 Institute of Cancer Prevention and Control, Hubei, China; 3 Maternal and Child Health Care Hospital, Wufeng, China

**Keywords:** Health economic evaluation, Cervical cancer, Screening, Markov model

## Abstract

**Background:**

Screening is the main preventive method for cervical cancer in developing countries, but each type of screening has advantages and disadvantages. To investigate the most suitable method for low-income areas in China, we conducted a health economic analysis comparing three methods: visual inspection with acetic acid and Lugol’s iodine (VIA/VILI), ThinPrep cytology test (TCT), and human papillomavirus (HPV) test.

**Methods:**

We recruited 3086 women aged 35–65 years using cluster random sampling. Each participant was randomly assigned to one of three cervical cancer screening groups: VIA/VILI, TCT, or HPV test. In order to calculate the number of disability-adjusted life years (DALYs) averted by each screening method, we used Markov models to estimate the natural development of cervical cancer over a 15-year period to estimate the age of onset and duration of each disease stage. The cost-effectiveness ratios (CERs), net present values (NPVs), benefit-cost ratios (BCRs), and cost-utility ratios (CURs) were used as outcomes in the health economic analysis.

**Results:**

The positive detection rate in the VIA/VILI group was 1.39%, which was 4.6 and 2.0 times higher than the rates in the TCT and HPV test groups, respectively. The positive predictive value of VIA/VILI (10.53%) was highest while the rate of referral for colposcopy was lowest for those in the HPV + TCT group (0.60%). VIA/VILI performed the best in terms of health economic evaluation results, as the cost of per positive case detected was 8467.9 RMB, which was 24503.0 RMB lower than that for TCT and 5755.9 RMB lower than that for the HPV test. In addition, the NPV and BCR values were 258011.5 RMB and 3.18 (the highest), and the CUR was 2341.8 RMB (the lowest). The TCT performed the worst, since its NPV was <0 and the BCR was <1, indicative of being poorly cost-beneficial.

**Conclusions:**

With the best economic evaluation results and requiring minimum medical resources, VIA/VILI is recommended for cervical cancer screening in poverty-stricken areas in China with high incidence of cervical cancer and lack of medical resources.

## Background

Cervical cancer is the third most common malignancy in women worldwide, with an estimated 528,000 new cases and 266,000 deaths in 2012 (approximately 85% of the global burden was from less developed countries) [1, 2]. Generally, cervical cancer incidence and mortality rates are highest in Eastern and Middle Africa [2]. Although the rates in China are better, there were still 62,000 new cases of cervical cancer in 2012; this is about five times greater than in the United States [2]. The incidence of cervical cancer in Wufeng County, a typical rural areas in China, is high (58.14 per 100,000), with a mortality rate of 22.43 per 100,000 in 2012, far above the Chinese average. More research is needed to effectively reduce the incidence of cervical cancer in the rural areas of China.

About 70% of cervical cancer cases are associated with human papillomavirus (HPV)-16, 18 [3]. This provides a new target for prevention. Since the first HPV vaccine was approved for use by the US Food and Drug Administration (FDA) in 2006, vaccination is rapidly becoming one of the main means of cervical cancer prevention [4]. However, HPV vaccines are not used worldwide. In fact, only six low-income countries have included HPV vaccination in their national immunization programs [5]. There are only three kinds HPV vaccines; these currently target subtypes 6/11/16/18/31/33/45/52/58, whereas HPV has up to 148 recognized subtypes [6–8]. HPV vaccine is recommended for girls aged 13–26 who have not been vaccinated [9, 10]. However, the World Health Organization (WHO) emphasizes that HPV vaccination should not replace cervical cancer screening, especially for those older than 26 years [11]. Therefore, screening remains essential for cervical cancer prevention globally, especially in developing countries.

Cervical cancer screening is more common than HPV vaccination in China. Since 2009, free cervical screening has been provided in some rural areas for women aged 35–59 years. However, in the first three years of the project, the coverage of women in rural areas was less than 2% [12]. Therefore, more efficient screening methods are needed. Three kinds of cervical screening methods (cytology, HPV test, and visual inspection with acetic acid [VIA/VILI] or with Lugol’s iodine [VILI]) are currently widely used [13]. Each of them has its advantages and disadvantages. In terms of cytology, the specificity of the traditional Pap smear in detecting cervical intraepithelial neoplasia (CIN) 2+ is high (96.3%), but sensitivity is low (53.0%); whereas the ThinPrep cytologic test (TCT) is more sensitive, but has the lowest specificity of all the screening methods. The sensitivity (96.1%) and specificity (90.7%) of the HPV test are satisfactory; however, it is a more difficult test to promote in low income areas because it requires more medical resources and is more costly (as is the TCT). The specificity (92%) and sensitivity (80%) of VIA are moderate, but it has a high false-positive rate and is highly subjective [14–16]. The screening organizer should take into account the applicability of each screening method, since the cost and outcome of the each will affect its use in specific places.

According to the WHO, less costly cervical cancer screening methods are more applicable in low income areas, when assessing cost only; not the combination of screening costs and outcomes [17]. In the present study, we conducted a health economic analysis to evaluate each cervical screening method in terms of combination of cost and outcome, to determine the most suitable screening method for poor rural areas in China where the incidence of cervical cancer is high.

## Method

This study was conducted in three parts: (1) three cervical cancer screening methods were applied to determine the costs and the positive cases in each step. (2) a Markov model was developed to estimate the natural development of cervical cancer for 15 years for the positive cases detected by screening. (3) a health economic analysis that included costing analysis, effectiveness/benefit/utility analysis, and cost-effectiveness/cost-benefit/cost-utility analysis was conducted, from the perspective of the user. In the study, the medical costs of screening were collected during screening. The unit costs of medical treatment were obtained from local the Maternal and Child Health Care Hospital. The daily social costs per person were obtained from the local “Statistical Yearbook (2014)”.

### Cervical cancer screening

Cluster random sampling was used to recruit 3086 women aged 35–65 years, as recommended by WHO and the Cancer Foundation of China (CFC) [18, 19]. Each subject was randomly assigned to one of three cervical cancer screening groups: VIA/VILI, TCT, or HPV test. Each screening method was performed according to the WHO’s “Comprehensive Cervical Cancer Control: A guide to essential practice (2nd edition)”. Cervical cancer was graded according to the International Federation of Gynecology and Obstetrics (FIGO) staging system (2009 version), and pathologic results were classified by the cervical precancerous lesions classification system released by the WHO in 2014. Women confirmed to be positive received appropriate treatment (surgery for ≥ CIN2, and follow-up for CIN1).

### Markov model application

**Fig. 1:**
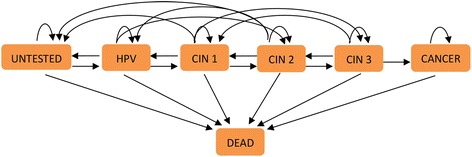
Mutual transition of the seven Markov states in the natural development of cervical cancer. A Markov cycle (one year) of mutual transition is described in the figure. The squares represent the seven different Markov health states. The arrows represent the direction of transition. The Markov state “CANCER” and “DEAD” cannot reverse transfer, and “DEAD” is the absorbing state, it cannot transfer to other states. HPV: HPV infection

We used TreeAge Pro 2011 software (TreeAge Software, Inc., Williamstown, MA, USA) to develop a Markov model to estimate the natural development of cervical cancer for 15 years (it takes approximately 10 years for precancerous cervical lesions to progress to cervical cancer, and death from cervical cancer is commonly expressed as a 5-year survival rate [19]). From this we estimated the age of onset and duration of each disease stage, in order to calculate the disability-adjusted life-years (DALYs) of each group. Seven Markov states were set up; the mutual transition of each Markov state is shown in Fig. 1.

Patients transitioned between the Markov states based on transition probabilities given in the following matrix [20–22]:

$$ \mathrm{P}=\left[\begin{array}{c}\hfill \begin{array}{ccc}\hfill 0.876\ \hfill & \hfill 0.123\hfill & \hfill 0.000\ \hfill \\ {}\hfill 0.300\ \hfill & \hfill 0.599\hfill & \hfill 0.086\ \hfill \end{array}\begin{array}{ccc}\hfill 0.000\hfill & \hfill 0.000\hfill & \hfill 0.000\hfill \\ {}\hfill 0.014\hfill & \hfill 0.000\hfill & \hfill 0.000\hfill \end{array}\begin{array}{c}\hfill\ 0.001\hfill \\ {}\hfill\ 0.001\hfill \end{array}\hfill \\ {}\hfill \begin{array}{ccc}\hfill 0.135\ \hfill & \hfill 0.015\hfill & \hfill 0.685\ \hfill \\ {}\hfill 0.120\ \hfill & \hfill 0.030\hfill & \hfill 0.140\ \hfill \end{array}\begin{array}{ccc}\hfill 0.149\hfill & \hfill 0.015\hfill & \hfill 0.000\ \hfill \\ {}\hfill 0.659\hfill & \hfill 0.050\hfill & \hfill 0.000\ \hfill \end{array}\begin{array}{c}\hfill 0.001\hfill \\ {}\hfill 0.001\hfill \end{array}\hfill \\ {}\hfill \begin{array}{ccc}\hfill 0.000\ \hfill & \hfill 0.000\hfill & \hfill 0.014\ \hfill \\ {}\hfill 0.000\ \hfill & \hfill 0.000\hfill & \hfill 0.000\ \hfill \\ {}\hfill 0.000\ \hfill & \hfill 0.000\hfill & \hfill 0.000\ \hfill \end{array}\begin{array}{ccc}\hfill 0.026\hfill & \hfill 0.919\hfill & \hfill 0.040\hfill \\ {}\hfill 0.000\hfill & \hfill 0.000\hfill & \hfill 0.761\hfill \\ {}\hfill 0.000\hfill & \hfill 0.000\hfill & \hfill 0.000\hfill \end{array}\begin{array}{c}\hfill\ 0.001\hfill \\ {}\hfill\ 0.239\hfill \\ {}\hfill\ 1.000\hfill \end{array}\hfill \end{array}\right] $$

Each column or row of the matrix represents a Markov state, and the numbers in each row are transition probabilities. More specifically, the first row is the transition probability that the state “UNTESTED” transfers to each other state (“UNTESTED”, “HPV”, “CIN 1”, “CIN 2”, “CIN 3”, “CANCER”, and “DEAD”) within a year. The second is the transition probability that the state “HPV” transfers to each other state, etc. After each annual cycle, patients either transitioned to a different Markov state or remained in the same state. We assumed that the state would stop processing after 15 years. To determine the number of patients in each state in the subsequent year, we multiplied the number of patient in each state by the corresponding transition probability. For example, the number of patients in the state “UNTESTED” multiplied by the probability in the first row gives the number of people in each state in the next year who transitioned from the state “UNTESTED”.

### Data analysis

#### Costing analysis

Cost can be divided into medical cost (medical resource consumption) and social cost (food, accommodation, transportation, communication and workforce productivity loss). The direct treatment cost of precancerous lesions and cervical cancer were specified by government (2400.0 RMB for CIN 2, 5650.0 RMB for CIN 3 and 8500.0 RMB for cancer). The radiotherapy and chemotherapy costs (54823.3 RMB) of cervical cancer were cited from relevant research [23]. Hospitalization data were provided by the local hospital. We averaged the hospital length of stay for the period 2011–2015, obtaining an average duration of hospitalization of 5.8 days for CIN 1, 7.5 days for CIN 2, 12.3 days for CIN 3, and 26.3 days for Cancer. From the social perspective, we obtained the daily per capita costs of diet (8.5 RMB), accommodation (3.7 RMB), transportation and communication (2.8 RMB), and wage (93.8 RMB) from the local “Statistical Yearbook (2014)”. Costs beyond the study duration were discounted at 3% per year. We multiplied the daily costs by the time spent on screening or hospitalization to calculate the social costs of screening and treatment.

#### Effectiveness, benefit and utility analysis

We used the positive detection rate, positive predictive value, and rate of referral for colposcopy as outcomes in the effectiveness analysis. The cost difference between the early-treated group and non-early treated group was calculated as the outcome in the benefit analysis. DALYs lost due to cervical cancer were used as the outcome in the utility analysis. The DALYs were calculated as the sum of the Years of Life Lost (YLL) due to premature death and the Years Lost due to Disability (YLD) for people living with the health condition or its consequences [24]. We calculated these using following equation derived by Murray [25, 26]:

$$ \mathrm{Y}\mathrm{L}\mathrm{L}\left(\mathrm{Y}\mathrm{L}\mathrm{D}\right)=-\frac{{\mathrm{DCe}}^{-\upbeta \upalpha}}{{\left(\upbeta +\upgamma \right)}^2}\left\{{\mathrm{e}}^{-\left(\upbeta +\upgamma \right)\mathrm{L}}\left[1+\left(\upbeta +\upgamma \right)\left(\mathrm{L}+\upalpha \right)\right]-\left[1+\left(\upbeta +\upgamma \right)\upalpha \right]\right\} $$

where D is the disability weight; γ (valued at 0.03) is the social discount rate; α is the age of the individual at the onset of symptoms; L is the duration of the disability or premature mortality; C (valued at 0.1658) is the age-weighting correction constant; and β (valued at 0.04) is the parameter from the age-weighting function.

The duration of the disability or premature death was derived from the results of Markov model simulation. The disability weight for precancerous lesions and cervical cancer were as follows: 0 for state less than “CIN 1”, 0.1238 for “CIN 2”, 0.1941 for “CIN 3”, 0.307 for “CANCER”, and 1for “DEAD” [27]. We used the model life table West level 26 for the standard life expectancy of women.

#### Economic analysis

We evaluated the cost-effectiveness of each screening method with cost effectiveness ratio (CER=cost/the number of positive patients) which was the cost of per positive patient been screened out. The net benefit value (NPV= benefit-cost), and benefit cost ratio (BCR= benefit/cost) were calculated in the cost-benefit analysis. Screening methods with a NPV>0 and BCR>1 were considered cost-beneficial. The cost utility ratio (CUR=cost/DALYs), that indicate the cost per DALY averted, was calculated in the cost-utility analysis.

#### Sensitivity analysis

Given the uncertainty about some parameters (radiotherapy and chemotherapy costs, discount rate) that were not collected during the study, univariate sensitivity analyses were used to assess the robustness of the health economic evaluation results. The variation in the range of radiotherapy and chemotherapy costs was ±5% and ±10% of the original value. The value of the discount rate changed by ±1% and ±2%.

## Results

### Cervical cancer screening results

**Fig. 2:**
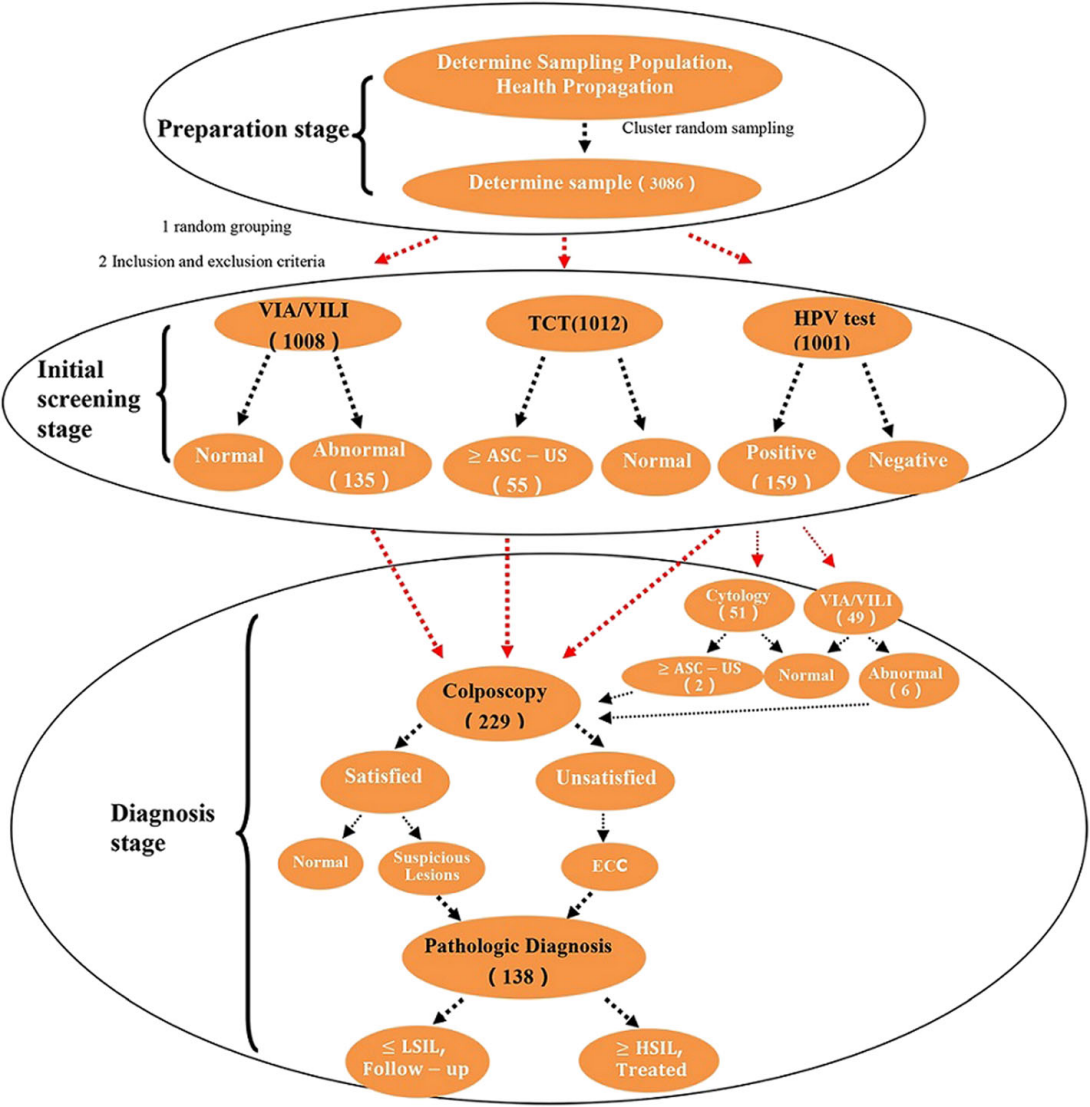
Cervical cancer screening procedure. Numbers in brackets denote the number of the procedure. Subjects identified as positive on initial HPV test screening were assigned to one of three re-examination groups (direct to Colposcopy, Cytology or VIA/VILI). Subjects who were positive in the initial VIA/VILI or TCT screening were re-examined directly by colposcopy. Endocervical curettage (ECC) was performed if colposcopy was inadequate or unsatisfactory

The detailed implementation procedure of cervical cancer screening is presented in Fig. 2. Fourteen positive cases were confirmed in the VIA/VILI group (8 had CIN 1, 3 had CIN 2, and 3 had CIN 3), three positive cases were confirmed in the TCT group (2 had CIN 1, and 1 had CIN3), and seven positive cases were confirmed in the HPV test group (2 had CIN 1, 1 had CIN 2, 3 had CIN3, and 1 had Cancer). The data of some subjects who were positive in the initial screening were missing from the re-examination of each group (2 from the VIA/VILI group, 12 from the TCT group, and 13 from the HPV test group).

### Health economic evaluation

#### Costing analysis

*Screening costs*: The total screening cost per capita was highest for VIA/VILI, followed by the HPV test (Table 1). However, the per capita initial screening costs of the three screening method were the opposite. This was due to the diagnosis confirmation costs, that were as high as 37367.5 RMB in the VIA/VILI group, 3.48 times that of TCT group and 2.34 times that of HPV test group. In total, the screening costs of the VIA/VILI group were highest (118550.6 RMB), and were 19338.0 RMB and 18983.8 RMB higher than those of the TCT group and the HPV group, respectively.

**Table 1:** Screening costs for the three screening groups

Cost components	VIA/VILI	TCT	HPV Test
Propaganda costs	51562.4 (51.2)	51562.4 (51.0)	51562.4 (51.5)
Initial screening costs			
Labor costs of drawing materials	12399.6 (12.3)	4145.1 (4.1)	4100.0 (4.1)
Consumable costs of drawing materials	5284.1 (5.2)	5088.2 (5.0)	5032.9 (5.0)
Instrument costs of drawing materials	446.4 (0.4)	448.2 (0.4)	443.3 (0.4)
Reading costs of TCT	–	23048.4 (22.8)	–
Detection costs of HPV test	–	–	13238.9 (13.2)
Costs of workforce productivity losses	5252.8 (5.2)	2157.4 (2.1)	2063.6 (2.1)
Confirmed diagnosis costs			
Colposcopy cost	13459.6 (101.2)	4352.9 (101.2)	5363.6 (101.2)
Costs of pathological examination	23908.0 (278.0)	6393.3 (278.0)	8062.0 (278.0)
VIA/VILI cost	–	–	877.1 (17.9)
TCT cost	–	–	1647.3 (32.3)
Costs of workforce productivity losses	6237.7 (46.9)	2016.7 (46.9)	7175.7 (46.9)
Total	118550.6 (117.6)	99212.6 (98.0)	99566.8 (99.5)

Data in the table represent the total cost (numbers outside parentheses) and per capita cost (numbers within parentheses). The unit for all values is RMB

*Treatment costs*: the direct medical costs accounted for the largest share of treatment costs, followed by indirect costs (Table 2). Thus, medical resource consumption was the main source of economic burden, with only a small portion of treatment cost accounted for by social costs. The early-treatment cost was highest for the HPV test group (97598.1 RMB). The non-early treatment cost was highest for the VIA/VILI group (413251.4 RMB); this cost was 1.20 times that of the HPV test group. The total treatment cost of the TCT group (68875.3 RMB) was the lowest.

**Table 2:** Treatment costs for the three screening groups

Cost (RMB)	VIA/VILI	TCT	HPV test
Early-treatment cost			
Direct medical cost	24150.0	5650.0	82673.3
Direct non-medical cost	1395.9	289.1	1661.5
Indirect cost	11143.4	2307.5	13263.3
Total	36689.3	8246.6	97598.1
Non-early treatment cost			
Direct medical cost	379939.8	63323.3	316616.5
Direct non-medical cost	3708.3	618.1	3090.3
Indirect cost	29603.3	4933.9	24669.4
Total	413251.4	68875.3	344376.2

Direct medical cost refers to the cost of medical resource consumption. Direct non-medical cost refers to the cost of diet, accommodation, transportation and communication. Indirect cost refers to the cost of workforce productivity losses. The non-early treatment cost was the cost of cervical cancer treatment for patients who were screened to be ≥ CIN2 (we assumed these patients did not receive intervention, and they developed a cervical cancer)

#### Effectiveness, benefit and utility analysis

**Fig. 3:**
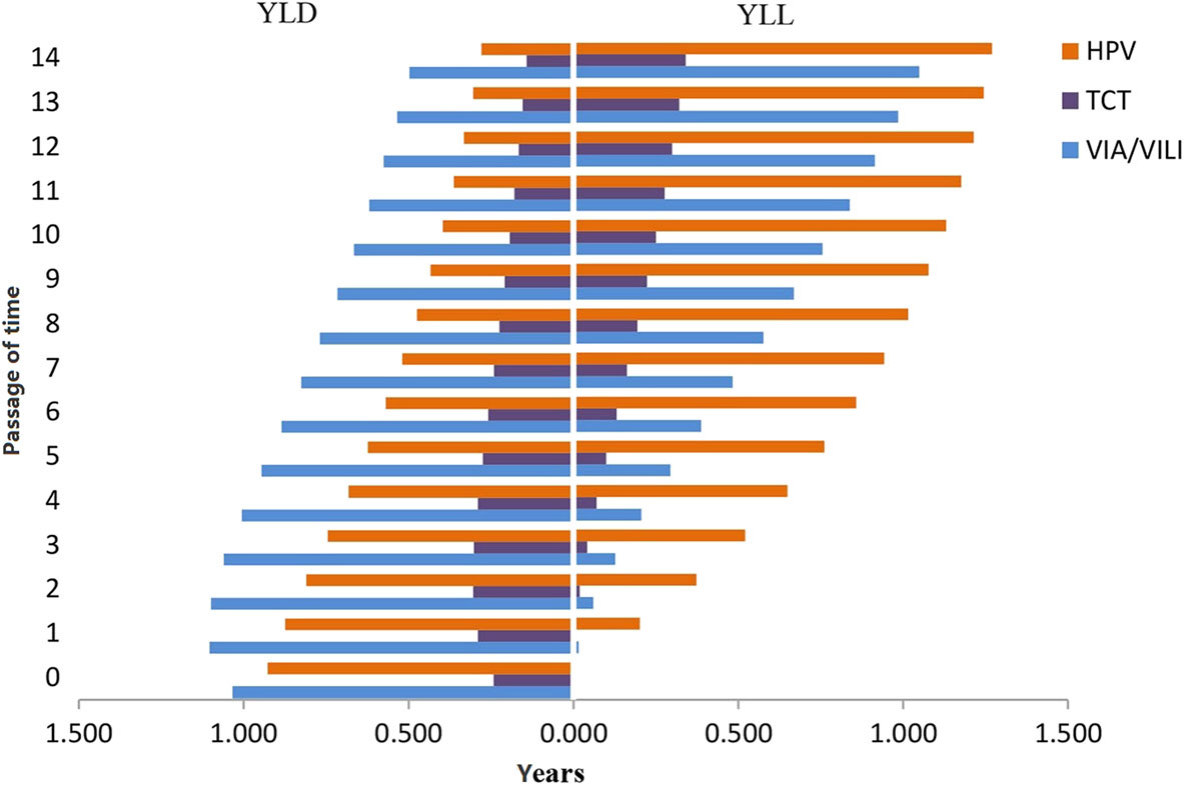
Simulation results of YLD and YLL averted by 3 screening methods with Markov model. Given the assumption that the state would not progress after 15 years, the DALYs averted in each group 14 years later were calculated in total. Thus, only the results for 14 years are shown. The YLD and YLL averted by each screening method 14 years later were 9.796, and 22.167 for VIA/VILI, 3.336, and 8.612 for TCT and 4.647, and 17.821 for the HPV test

*Simulation results of Markov model*: according to the simulation results of natural development of cervical cancer for 15 years, VIA/VILI averted the greatest number of YLD every year, followed by the HPV test (Fig. 3). However, the YLL averted by these two methods was opposite. The TCT had the fewest YLD and YLL averted. The YLD averted by the three screening methods generally decreased over time, while the YLL increased over time.

*Effectiveness*: both the positive detection rate (1.39%) and the positive predictive value (10.53%) were higher in the VIA/VILI group than in the other two groups (Table 3). The rate of referral for colposcopy in the HPV test + TCT group (0.60%) was lowest, and HPV test + VILI (1.80%) was second. *Benefit*: the economic benefits brought by the VIA/VILI were 376562.1 RMB, much higher than the other two. *Utility*: The DALYs averted by VIA/VILI (50.624), was the highest of all the groups.

**Table 3:** Economic analysis of the three screening groups

Group	Cost (RMB)	Cost-effectiveness				Cost-benefit			Cost-utility	
		Positive detection rate (%)	Positive predictive value (%)	Referral rate of colposcopy (%)	CER (RMB)	Benefit (RMB)	NPV (RMB)	BCR	DALYs	CUR (RMB)
VIA/VILI	118550.6	1.39	10.53	13.19	8467.9	376562.1	258011.5	3.18	50.624	2341.8
TCT	99212.6	0.30	6.98	4.25	33070.9	60628.7	−38583.9	0.61	17.603	5636.1
HPV test	99566.8	0.70	4.80	5.29	14223.8	246778.1	147211.3	2.48	42.301	2353.8

#### Economic analysis

The value of a screening program is influenced by both input and output. Although the per capita costs of VIA/VILI were highest, the cost-effectiveness (8467.9 RMB per person for CER), cost-benefit (258011.5 RMB for NPV and 3.18 for BCR), and cost-utility (2341.8 RMB per DALY averted for CUR) of VIA/VILI were all optimal (Table 3). In terms of the cost-utility analysis, there was little difference between the HPV test group and the VIA/VILI group, but the results of the cost-effectiveness analysis and cost-benefit analysis were markedly different. TCT had the worst results in the economic analysis with a NPV <0 and a BCR <1, which indicate a poor cost-benefit.

#### Sensitivity analysis (Fig. 4)

When the variation range of radiotherapy and chemotherapy costs were ±5 and ±10% of the original value, the CER and CUR of three groups were unaffected, indicating good stability. However, the NPVs and BCRs were influenced greatly, such that the maximum range of variation of NPV was up to ±14.9% of the original value, and the maximum range of variation of BCR was up to ±9.24% of the original value. When the value of the discount rate changed ±1% and ±2%, the results of economic evaluation were very stable; the maximum variation range was only 0.19%. In all, the results of the comparison of the three screening groups for economic evaluation did not change, irrespective of the cost of radiotherapy or chemotherapy. Similarly, whether the discount rate changed or not, the CER, NPV, BCR, and CUR values of VIA/VILI were still the best of the three groups.

**Fig. 4:**
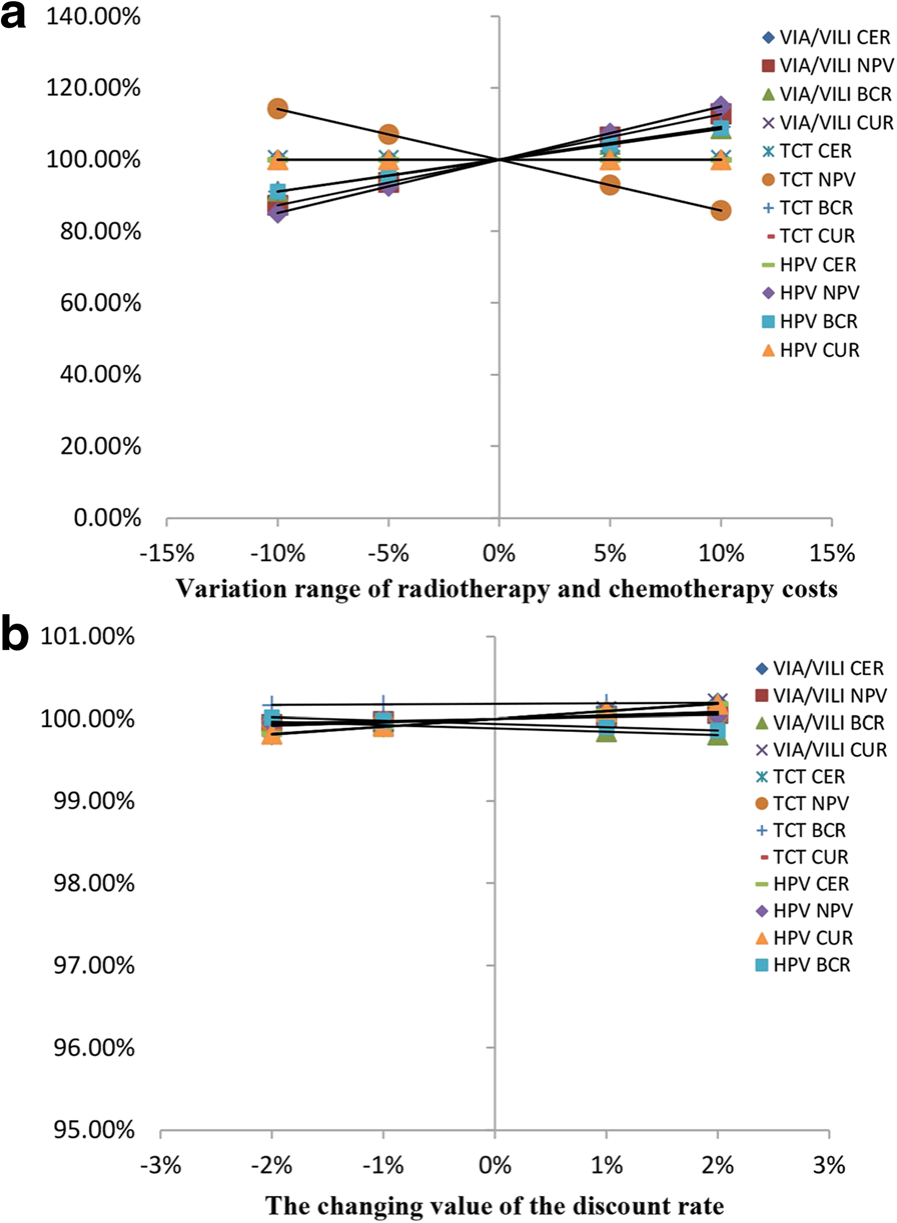
Sensitivity analysis for the three screening groups. “**a**” indicates the results of economic evaluation changes with radiotherapy and chemotherapy costs. “**b**” indicates the results of economic evaluation changes with discount rate. The ordinates in “**a**” and “**b**” represent ratios of the economic evaluation index before and after changes. In figure “**a**” and “**b**”, the smoother the straight line, the more stable the result

## Discussion

The economic burden of HPV vaccination in rural areas is unaffordable; cervical cancer screening is still the primary preventive measure for cervical cancer. Our findings show that the per capita treatment cost of cervical cancer was 63323.3 RMB. However, the annual net income per capita was 8895.9 RMB in rural areas of China, with only 2270.4 RMB of disposable income when excluding life consumer spending [28]. Despite the Chinese government’s establishment of the New Rural Cooperative Medical System for treatment of serious illnesses in patients in rural areas, under which up to 70% of the cost of hospitalization for cervical cancer is reimbursed, the economic burden of the remaining costs of cervical cancer treatment (18997.0 RMB) for rural women has reached 213.6% [29]. Although both HPV vaccination and cervical cancer screening are ways to prevent cervical cancer, the economic burden of a single dose of HPV vaccine is 7.12% (63.3 RMB), while the maximum economic burden of cervical cancer screening (not including the cost of educational material/advertising) is 0.75% (66.4 RMB) [30].

The averted DALYs of each patient in this study were calculated more specifically, as a combination of the Markov model and calculation formula of DALYs, derived by Murray, was performed. This was weighted by age, and different stages of the disease were included in the calculation. As far as we know, this is the first time these two methods for calculating DALYs have been used in combination in cervical cancer research. A search for previous studies on cervical cancer in the PubMed database identified only three studies with similar detailed handling (such as age-weighting and including different disease stages) of DALY calculation. Only two were related to health economic evaluation, and none combined the Markov model with the DALY calculation formula derived by Murray [31–33].

The cost-utilities of VIA/VILI, TCT, and the HPV test, especially for VIA/VILI, were very effective in the study, since the CURs were lower than China’s GDP per capita (GDP per capita = 49886.8 RMB in 2014, according to the World Bank [34]). However, this result is based on a high participation rate in cervical cancer screening programs. The participation rate in this study was 97.9%, whereas studies on cervical cancer screening in rural areas of China in recent years have shown a participation rate of approximately 24% [28, 35]. Even in rural areas where cervical cancer screening is free of charge, the participation rate in VIA/VILI screening programs can be as low as 35% [35]. Thus, improvement of participation rates among women in rural areas is necessary before implementation of cervical cancer screening.

The VIA/VILI is more suitable for cervical cancer prevention in the rural areas that lack medical resources and a high incidence of cervical cancer than TCT or HPV test; it showed the best economic evaluation results and requires minimal medical resources. Consistent with this finding, the WHO proposed that VIA/VILI should be the first choice for areas with a shortage of medical resources [17]. Despite the advantages of VIA/VILI, its sensitivity and specificity are vulnerable to the subjective understanding and clinical experience of screening operators. The specificity of VIA/VILI can be as high as 90% if screening operators have been professionally trained [36]. Therefore, more attention should be paid to the training of operators performing cervical cancer screening.

There are some limitations to this study. First, because of the limited medical resources in the study area, hospitals lacked outpatient medical record systems, making it difficult to collect clinical data. Second, only the expenses of hospitalization were included in direct medical treatment costs. Last, since the use of several joint screening methods has been proposed, the use of the three cervical cancer screening methods independently in the study could be a limitation.

## Conclusion

VIA/VILI is recommended for cervical cancer screening in rural areas with a high incidence of cervical cancer. It is a more effective method of reducing the burden of cervical cancer considering the local economic level and requires less medical equipment and personnel than the other two screening methods. Future studies on health economic evaluation of cervical cancer screening in rural areas should take clinic data into account or focus on combined screening.

## Abbreviations

BCR: Benefit-cost ratio
CER: Cost-effectiveness ratio
CUR: Cost-utility ratio
DALY_S_: Disability-adjusted life years
ECC: Endocervical curettage
HPV: Human papillomavirus
NPV: Net present value
TCT: ThinPrep cytology test
VIA/VILI: Visual inspection with acetic acid and Lugol’s iodine
YLD: Years lost due to disability
YLL: Years of life lost

## References

[1] Arbyn M, Castellsague X, de Sanjose S, Bruni L, Saraiya M, Bray F (2011). Worldwide burden of cervical cancer in 2008. Ann Oncol.

[2] World Health Organization, International Agency for Research on Cancer. http://globocan.iarc.fr/Pages/fact_sheets_cancer.aspx. Accessed 25 Apr 2016.

[3] Forman D, de Martel C, Lacey CJ, Soerjomataram I, Lortet-Tieulent J, Bruni L (2012). Global burden of human papillomavirus and related diseases. Vaccine.

[4] Bosch FX, Broker TR, Forman D, Moscicki AB, Gillison ML, Doorbar J (2013). Comprehensive control of human papillomavirus infections and related diseases. Vaccine.

[5] Garland SM, Smith JS (2010). Human papillomavirus vaccines: current status and future prospects. Drugs.

[6] Bernard HU, Burk RD, Chen Z, van Doorslaer K, Zur HH, de Villiers EM (2010). Classification of papillomaviruses (PVs) based on 189 PV types and proposal of taxonomic amendments. Virology.

[7] Boiron L, Joura E, Largeron N, Prager B, Uhart M (2016). Estimating the cost-effectiveness profile of a universal vaccination programme with a nine-valent HPV vaccine in Austria. BMC Infect Dis.

[8] Gattoc L, Nair N, Ault K (2013). Human papillomavirus vaccination: current indications and future directions. Obstet Gynecol Clin North Am.

[9] Centers for Disease Control (2010). FDA licensure of bivalent human papillomavirus vaccine (HPV2, Cervarix) for use in females and updated HPV vaccination recommendations from the Advisory Committee on Immunization Practices (ACIP). MMWR Morb Mortal Wkly Rep.

[10] Markowitz LE, Dunne EF, Saraiya M, Lawson HW, Chesson H, Unger ER (2007). Quadrivalent human papillomavirus vaccine: recommendations of the advisory committee on immunization practices (ACIP). MMWR Recomm Rep.

[11] World Health Organization. WHO Position Paper on Vaccines against Human Papillomavirus (HPV). 2014. http://www.who.int/immunization/position_papers/pp_hpv_oct2014_presentation.pdf. Accessed 25 Apr 2016.

[12] The Lancet. The Lancet (2009) Women’s health in rural China. Lancet. 2009;374(9687):358. http://dx.doi.org/10.1016/S0140-6736(09)61394-5. 10.1016/S0140-6736(09)61394-5 19647592

[13] World Health Organization. Screening for cervical cancer. 2016. http://www.who.int/cancer/detection/cervical_cancer_screening/en/. Accessed 25 Apr 2016.

[14] Cuzick J, Clavel C, Petry KU, Meijer CJ, Hoyer H, Ratnam S (2006). Overview of the European and North American studies on HPV testing in primary cervical cancer screening. Int J Cancer.

[15] Sauvaget C, Fayette JM, Muwonge R, Wesley R, Sankaranarayanan R (2011). Accuracy of visual inspection with acetic acid for cervical cancer screening. Int J Gynaecol Obstet.

[16] Arbyn M, Bergeron C, Klinkhamer P, Martin-Hirsch P, Siebers AG, Bulten J (2008). Liquid compared with conventional cervical cytology: a systematic review and meta-analysis. Obstet Gynecol.

[17] World Health Organization, Regional Office for South-East Asia. Strategic framework for the Comprehensive Control of Cancer Cervix in South-East Asia Region. 2015. http://www.who.int/iris/handle/10665/152098. Accessed 30 Apr 2016.

[18] Dong Z-W (2005). Chinese guidelines for screening and treatment of precancerous lesions for cervical cancer prevention.

[19] World Health Organization. Comprehensive Cervical Cancer Control: A guide to essential practice (Second edition). http://apps.who.int/iris/bitstream/10665/144785/1/9789241548953_eng.pdf?ua=1. Accessed 30 Apr 2016. 25642554

[20] Nasiell K, Roger V, Nasiell M (1986). Behavior of mild cervical dysplasia during long-term follow-up. Obstet Gynecol.

[21] Denny L, Kuhn L, Pollack A, Wright TJ (2002). Direct visual inspection for cervical cancer screening: an analysis of factors influencing test performance. Cancer.

[22] Li G-R (2010). Application of Markov model in performance evaluation of cervical cancer screening. Chin J Health Stat.

[23] Xubin Bai. Economic Evaluation of the VIA/VILI Program--Early Diagnosis and Early Treatment of Cervical Cancer in the Countryside of Shanxi province. Taiyuan: Shanxi Medical University; 2009.

[24] World Health Organization. Health statistics and information systems: Disability-Adjusted Life Year (DALY). 2016. http://www.who.int/healthinfo/global_burden_disease/metrics_daly/en/. Accessed 26 Apr 2016.

[25] Murray CJ (1994). Quantifying the burden of disease: the technical basis for disability-adjusted life years. Bull World Health Organ.

[26] LA Murray CJL (1996). The global burden of disease: a comprehensive assessment of mortality and disability from disease, injuries, and risk factors in 1990 and projected to 2020.

[27] Tang X, Qiao Y-L, Li G-R (2010). Study on the method of determining the weight values in the calculation of DALYs. Chin J Health Stat.

[28] National Health and Family Planning Commission of the People’s Republic of China (2016). Chinese Health and family planning statistical yearbook (2015).

[29] Zeng M (2014). Practice and thinking: hospital over the treatment and aid of a serious illness by NRCMS--Two cancer in women (breast cancer, cervical cancer), for example. Chin Health Ind.

[30] Liu Y-J, Zhang Q, Hu S-Y, Zhao F-H (2016). Effect of vaccination age on cost-effectiveness of human papillomavirus vaccination against cervical cancer in China. BMC Cancer.

[31] Salomon JA, Carvalho N, Gutierrez-Delgado C, Orozco R, Mancuso A, Hogan DR (2012). Intervention strategies to reduce the burden of non-communicable diseases in Mexico: cost effectiveness analysis. BMJ.

[32] Ginsberg GM, Lauer JA, Zelle S, Baeten S, Baltussen R (2012). Cost effectiveness of strategies to combat breast, cervical, and colorectal cancer in sub-Saharan Africa and South East Asia: mathematical modelling study. BMJ.

[33] Hristina V, Sandra Š-G, Slavenka J, Jelena M, Nikola K, Ljiljana M-D (2006). Burden of Cancer in Serbia. Croat Med J.

[34] the World Bank. GDP per capita (current US$):World Bank national accounts data, and OECD National Accounts data files. http://data.worldbank.org/indicator/NY.GDP.PCAP.CD?locations=CN&view=chart Accessed 30 Apr 2016.

[35] National Health and Family Planning Commission of the People’s Republic of China. Report: Women and children benefit from the deep medical reform. 2014. http://www.nhfpc.gov.cn/fys/gzbs/201405/89b14a1d2b3348a88abce88863855a72.shtml Accessed 21 Nov 2016.

[36] Mahe C, Gaffikin L (2005). Screening test accuracy studies: how valid are our conclusions? Application to visual inspection methods for cervical screening. Cancer Causes Control.

